# Behavioral and Chemical Ecology of Marine Organisms with Respect to Tetrodotoxin

**DOI:** 10.3390/md8030381

**Published:** 2010-02-26

**Authors:** Becky L. Williams

**Affiliations:** Department of Biology, MSC 3AF, New Mexico State University, PO Box 30001, Las Cruces, New Mexico, 88003-8001, USA; E-Mail: toxwilliams@gmail.com; Tel.: +01-1-575-646-4123; Fax: +01-1-575-646-5665

**Keywords:** tetrodotoxin, ecology, defense, venom, pheromone

## Abstract

The behavioral and chemical ecology of marine organisms that possess tetrodotoxin (TTX) has not been comprehensively reviewed in one work to date. The evidence for TTX as an antipredator defense, as venom, as a sex pheromone, and as an attractant for TTX-sequestering organisms is discussed. Little is known about the adaptive value of TTX in microbial producers; thus, I focus on what is known about metazoans that are purported to accumulate TTX through diet or symbioses. Much of what has been proposed is inferred based on the anatomical distribution of TTX. Direct empirical tests of these hypotheses are absent in most cases.

## 1. Introduction

### 1.1. Overview and Justification

Tetrodotoxin (TTX) is a small molecular weight guanidinium neurotoxin that occludes voltage-gated sodium channels in nerve and muscle tissue thereby impeding ion conductance [1,2]. Named for the family of pufferfish in which it was first discovered (Tetrodontidae), TTX and a bevy of less toxic analogs [3–7], have been documented from many disparate taxa [8–11]. Given the myriad studies on the taxonomic and anatomical distribution, physiological action, and medical uses of TTX, the paucity of information on the ecological function of TTX is striking. Although many reviews cover the occurrence, physiological action, and production of TTX (>120 on Web of Science), the ecology of TTX-bearing organisms is not the main focus and is not thoroughly covered in any one work to date. The ecology of terrestrial organisms involving TTX is discussed elsewhere [12,13] and will only be briefly mentioned in this work. Here I review the behavioral and chemical ecology of marine organisms mediated by TTX to emphasize gaps in knowledge, as much as to compile the available literature, in hopes of spurring future research on this subject.

### 1.2. Background

The multi-ring structure in this small molecule [14–17], the difficulty in identifying a natural biosynthetic pathway [18–23], and the stereospecificity of TTX [24] suggest this toxin is unlikely to arise by convergent pathways. Instead, the prevailing theory is that TTX is produced by several species of bacteria, both free-living and symbiotic [10,11,25]. Many marine natural products ultimately derive from the diet or symbiotic microorganisms [26–28]. Determining the ultimate source of TTX is critical for evaluating the role of TTX in the ecology of TTX-bearing organisms because the strength of selection may be attenuated if filtered through exogenous sources. For example, populations of organisms that accumulate toxins from their diet may not be able to respond to the selection pressure of predation by increasing their own toxin content if dietary sources are limited. Although insect herbivores that sequester toxin defenses from plant hosts may target and increase uptake of co-opted defenses [29], and similar behavioral preferences may be in place for TTX-accumulating organisms [30,31], the toxin content of these organisms could still be limited by available resources.

Matsumura [32,33] criticized much of the research identifying TTX-producing bacteria because of a lack of complete controls and non-specific techniques used to identify TTX in bacterial cultures. Matsumura [33,34] observed that the polypeptone media and yeast extracts used to grow some cultures caused false positives for TTX in both the standard HPLC and GC-MS methods used for TTX identification. I have similarly observed confounding small molecular weight constituents in snake musk that prevented identification of TTX via HPLC [34]. Additionally, the presence of multiple, potentially toxic, constituents in some bacterial extracts obfuscates the specificity of the mouse bioassay also used to quantify and confirm the identity of TTX [32,33]. The same issues of non-specificity have been raised about the identification of saxitoxin (STX), which is structurally similar to TTX [35]. Matsui *et al.* [36] claimed to transform a non-toxic cultured puffer fish (*Takifugu rubripes*) by infecting it with TTX-producing bacteria (*Shawanella putrefaciens*) provided in commercial food. However, of multiple subjects, only one fish appeared to acquire TTX in the liver. The identification methods were HPLC and the mouse bioassay and control chromatograms of TTX-free liver and authentic TTX were not provided for comparison in that brief note [36]. While the result is intriguing, confirmation with further infection experiments would be valuable.

Confirmation through full biochemical characterization or via monoclonal antibodies to TTX [37–47] present potential solutions to these problems identifying TTX. Production of TTX in at least one bacterium (*Pseudoalteromonas haloplanktis tetraodonis*) was confirmed by immunochemistry with a TTX-specific antibody [47]. Because many bacteria are strongly suggested to produce TTX through multiple lines of evidence [10,11,25], bacterial production of the closely related saxitoxin (STX) has been confirmed, and the STX pathway can be shared via horizontal gene transfer with dinoflagellate hosts [49,50], the bacterial production of TTX seems plausible in some cases. However, weak chemical traces or lack of correlation of proposed TTX quantities and toxicity [51,52] call for re-characterization of TTX production in other purported bacterial sources [32,33].

Some researchers suggest an endogenous origin of TTX in a few taxa as a viable alternative to bacterial symbiont and dietary accumulation hypotheses. After fertilizing eggs in artificial seawater in the laboratory, Matsumura [44] detected an increase in TTX in puffer fish (*Takifugu niphobles*) embryos during development before hatching. Research on TTX-bearing terrestrial newts confounds the picture further. Lehman *et al.* [53] found no evidence of bacterial symbionts (mtDNA signatures) coincident with TTX stores in one species of terrestrial newt (*Taricha granulosa*) [53]. The same species increases TTX levels in captivity with a TTX-free diet, and even regenerates levels of TTX purged after expulsion via electric shock [54,55]. However, another TTX-bearing newt (*Cynops pyrrhogaster*) loses TTX stores after maintenance in captivity and demonstrates the ability to accumulate TTX when offered TTX-laden food [10,56]. Thus, additional work on the ultimate origin of TTX is called for in some taxa and broad generalizations should be viewed with caution. Discovery of the biosynthetic pathway will be critical to resolving the controversy surrounding the ultimate origin of TTX [18,28].

In TTX-bearing taxa the possibility of accumulation is afforded by the physiological resistance of the organism to the toxic effects of TTX. Resistance to TTX is often conferred by changes in the binding affinity of TTX to the target sodium channels, and several taxa have independently converged on this solution [57–59]. Alterations of a single amino acid at the external sodium channel pore can eliminate interaction with specific polar groups on the TTX molecule, and changes at multiple binding sites confer cumulative resistance [60–62]. In contrast, resistance in shorecrabs (*Hemigrapsus sanguineus*) is attributed to a binding protein in the hemolymph [63]. Once resistance to TTX occurs, the stage is set for an organism to accumulate vast amounts of the toxin via symbionts or diet and to potentially co-opt TTX for an evolutionary leg-up over their non-toxic counterparts. Sequestration of dietary compounds occurs in many marine organisms, which deploy a cocktail of these chemicals for their own defense as well as other ecological functions [26,27].

In puffer fishes, bioaccumulation from the diet is implicated as an important component to toxification in conjunction with TTX-producing symbionts. Some species (*Takifugu poecilonotus*, *T. niphobles*) rapidly accumulate the toxin from their diet when offered TTX-laden food items [64–71], and another species (*T. rubripes*) preferentially accumulates TTX in its liver over the closely related STX [69]. Additionally, puffer fishes (*T. rubripes, T. niphobles*) raised in captivity have undetectable levels of TTX [66,72–74], though a more sensitive analysis in one study revealed low levels [75]. The accumulation of TTX in the liver of *T. rubripes in vitro* is facilitated by a carrier molecule, which indicates active sequestration of TTX [74–75]. Other binding proteins in the plasma and liver of puffer fishes (*T. pardalis*, *T. niphobles*, *T. poecilonotus*) [72,78–80] and the hemolymph of a shorecrab (*Hemigrapsus sanguineus*) [63] have been identified. Non-TTX-resistant fish do not sequester TTX in the same manner and instead excrete or metabolize small amounts of the toxin (higher concentrations can be fatal) [76–77]. Finally, a starfish (*Astropecten scoparius*) also appears to accumulate TTX from bivalve and gastropod prey (*Veremolpa scabra* and *Umborium suturale*) [81].

### 1.3. Objective

Regardless of the origin, whether endogenous, via dietary bioaccumulation, or via symbionts, TTX is assumed to benefit accumulators in many cases. There can be physiological costs associated with TTX resistance [82,83], and the cost of maintaining TTX-producing bacteria may be significant, as it is in other mutualistic relationships [84,85]. Thus, the benefit of accumulating vast amounts of TTX must exceed that of excreting or destroying it. Unfortunately, given the debate surrounding TTX biosynthesizers, the adaptive value of TTX in original producers is largely unknown. Tetrodotoxin does not appear to be a side product of any other known pathway and its complicated structure indicates an involved biosynthesis [18–23] that presumably imposes a substantial cost. While the possibility exists that TTX accumulating organisms do not benefit from their stores, the evidence of TTX sequestration in some taxa indicates otherwise. Tetrodotoxin is water-soluble and thus relatively easily excreted [8]; there should be no inherent advantage in producing binding proteins if excretion were the only advantageous outcome. Thus, the extreme longevity of TTX in tissues of several organisms [34,67] and the presence of binding proteins specific for TTX [63,72,76–80] suggest that these taxa do benefit from its presence. Much of the functionality in accumulators is assumed based on distribution of TTX in tissues, but some researchers have empirically tested the benefit of TTX. The following synopsis covers proposed or discovered defensive, offensive, and communicative functions of TTX in TTX-bearing organisms.

## 2. Defensive Function of TTX (Antipredator)

The most common (and intuitive) assumption is that TTX serves an antipredator function for organisms that produce or accumulate it. However, this assumption has only rarely been empirically tested with potential predators. Even obvious assumptions are sometimes incorrect and actual quantification of the value of the assumed defense (e.g., covariance of survival probability with TTX quantities) is clearly important to gauge the effect TTX will have on interspecies interactions. While a suggested function of TTX in one Planocerid worm was antipredator defense [86], this assumption was not borne out. Actual predators consumed another TTX-bearing Planocerid with apparent impunity [87]. Instead, the worm employs TTX as venom to subdue its own prey [87]. However, in the absence of empirical data, educated hypotheses about the role of TTX in antipredator defense must be inferred.

The occluding effect of TTX on vertebrate sodium channels is well characterized [1,2]. Whether TTX confers protection to free-living bacterial producers from their non-vertebrate predators is unknown. The evidence that TTX appears to be an endproduct, rather than a biproduct of another biosynthetic pathway [18–23], and that it is purportedly produced by a wide variety of free-living bacteria [88–91] imply an important function in microbial producers. Though little information on the adaptive value of TTX is available for microbial producers given the lingering controversy on TTX biogenesis, at least some information on the closely related saxitoxin (STX) is available. Saxitoxin and other paralytic shellfish poisons in dinoflagellates and cyanobacteria inhibit grazing by copepods and ciliates––or even cause cell lysis [13,92]. However, different strains of dinoflagellates exhibit differing toxicities such that some copepods are not deterred by some strains [93] and some amphipods are actually stimulated by STX [94]. Clearly, much work on the adaptive value of TTX in free-living microbial producers is needed.

In TTX accumulating organisms, co-opted function of TTX can be partially inferred by the distribution in tissues. For example, TTX occurs in the skin of several organisms such as puffer fishes, amphibians, blue-ringed octopuses, and gobies [10]. In puffer fishes, immunolabeling studies revealed TTX in skin glands of *Takifugu vermicularis*, skin succiform cells of *Chelonodon patoca*, and undifferentiated basal cells and succiform cells in the skin of *Tetraodon steindachneri* [41,42,46]. Other puffer fishes (*Takifugu pardalis*, *T. niphobles*, *T. poecilonotus, T. vermiculare radiatum*, and *T. vermiculare porphyreum*) harbor TTX in exocrine glands (or gland-like structures) in the skin, which is excreted upon electrical stimulation [64,95]. In contrast, *T. xanthopterum* and *T. rubripes* do not excrete TTX upon electrical stimulation [64]. Saito *et al.* [96] found that *T. niphobles* as well as *T. v. vermicularis* and *T. pardalis* release large quantities of TTX from the skin upon handling stimulus. Additionally, the majority of TTX administered intramuscularly to *T. rubripes* is accumulated in the skin within 12 h [97]. The excretion of TTX from the skin of puffer fishes is often, but not always, associated with inflating [64,95,96], a common response to predators [98].

Notably, plastic models of the puffer fish (*Canthigaster valentini*) repel predators and even support a Batesian mimic (*Paraluteres prionurus*) [99,100]. Protection is presumably conferred by TTX, though this species has not been assayed for TTX and the specific antipredator contribution of TTX in this system was not explicitly tested. Shimizu *et al.* [101] also found that hatchery raised puffer fish (*T. rubripes*) succumbed to predation more often than wild juveniles. They attributed the higher mortality to maladaptive swimming behavior and lower (undetectable) TTX concentration in the hatchery juveniles compared to the wild fish [101]. Again, a direct correlation between TTX stores and survival probability was not tested.

The snail, *Natica lineata*, stores TTX in its muscle [102] and absorbs water into the “muscle cavity”, which it releases loaded with TTX upon disturbance [103]. Another gastropod, *Niotha clathrata*, also secretes TTX when stimulated [104]. A ribbonworm (*Cephalothrix* spp.) harbors TTX in the bacillary cells of the epidermis [47], and at least one species (*Cephalothrix linearis*) excretes TTX upon stimulation [105]. Blue-ringed octopuses (*Hapaloclaena fasciata*, *H. lunulata*, and *H. maculosa*) also harbor tetrodotoxin in the skin [106,107]; however, external secretion has not been reliably demonstrated. An antipredator function is suggested by the presence of seemingly aposematic blue rings and the finding that the greatest overall quantities of TTX reside in the skin [106,107]. The goby (*Yongeichthys criniger*) also harbors disproportionately high TTX stores in the skin [108]. Finally, several terrestrial amphibians also contain TTX in the skin [109], and TTX can be associated with granular glands that typically function in toxin excretion [110,111].

Furthermore, human intoxications from consumption of puffer fish, snails, and crabs are not uncommon [10]. Thus, the poisonous nature of many TTX-accumulating organisms is apparent. The distribution of TTX in skin, and skin glands in some cases, in conjunction with secretion during stimulation and human intoxications strongly implies an antipredator function for TTX in these taxa; however, empirical experiments with natural predators directly relating survival probability to TTX stores are lacking for any of these species. Given the potential range of predator responses to a single chemical, such natural history investigations are critical to evaluating the realized antipredator efficacy of a defense in wild populations.

The ovaries of many puffer fishes often harbor the highest levels of TTX [10]. Tetrodotoxin also occurs in the oocytes of the puffer fishes *T. vermicularis* and *Chelonodon patoca* [42], and the eggs of *T. niphobles* [44,112]. Other taxa harbor TTX in the eggs as well, including a Planocerid flatworm [86], a horseshoe crab (*Carcinoscorpius rotundicauda*) [113,114], the flatworm (*Planocera multitentaculata*) [86], and at least one species of blue-ringed octopus (probably *Hapalochlaena fasciata* based on locality) [115]. Tetrodotoxin was also localized in the ovum of the flatworm, *Planocera reticulata*, and the ribbonworm, *Cephalothix* sp. [47]. The larvae and eggs of the sharpnose puffer fish (*Canthigaster valentini*) deterred 6 species of reef fishes, which sampled, but ultimately rejected, the eggs and larvae [116]. The larvae appeared to escape unharmed. Gladstone [116] assumed the distasteful substance was TTX, but this was not empirically tested. Neither the eggs or larvae were examined for TTX, nor were otherwise palatable food items spiked with TTX offered to verify noxiousness. One congener (*C. bennetti*) ingested the larvae of *C. valentini* with no ill effects [116] and other congeners are known to contain TTX [117,118], but the deterrence still cannot be definitively assigned to TTX. Thus, no empirical tests have unequivocally demonstrated an antipredator function of TTX in eggs from any marine organism.

Interestingly, nervous stimulation of some fishes in response to TTX has been characterized directly. Both TTX and STX stimulated an electrical response downstream of tastebuds of rainbow trout (*Oncorhynchus mykiss*) and arctic char (*Salvelinus alpinus*); the authors hypothesize this sensitivity helps these fishes avoid toxic prey items [119–121]. Gustatory receptors for TTX are independent of those for STX in these species [120]. Yamamori *et al.* [120] also cite their work that demonstrates fish rejecting food items tainted with TTX; unfortunately, this research does not appear to have been published. Again, although this research is very suggestive that TTX may be deterrent to some fish, the empirical confirmation is lacking.

The presence of TTX was discovered in the ink of 3 out of 7 blue-ring octopus (*Hapalochlaena lunulata*), including in one individual that did not have detectable TTX in the putative source organ—the posterior salivary gland [106]. This may indicate a potential antipredator function, given that inking is a common defense mechanism of mollusks [27], but has not been tested. Finally, some observations about the antipredator function of the closely related STX are again relevant. Bioaccumulation of STX in butter clams (*Saxidomus giganteus*) appears to afford some protection from otters (*Enhydra lutris*) [122]. When STX levels are high, otters switch to smaller, less common, less toxic shellfish prey and larger butter clams occur at higher densities [122]. Parallel behavioral and natural history fieldwork required for the verification of the proposed antipredator hypotheses for TTX-bearing organisms is lacking.

## 3. Offensive Function of TTX (Venom)

Although the function of TTX in most organisms appears to be defensive, at least a few species may exploit TTX to subdue their own prey. One species of Planocerid flatworm appears to use TTX to overcome their much larger gastropod prey [87]. The localization of TTX and 11-norTTX-6(*S*)-ol in the pharynx combined with a decrease in these toxins after feeding indicates that TTX was introduced into the prey items during attack or feeding [87]. After an additional week, toxin stores were replenished in the worms. Additionally, several species of poison arrowworms (Chaetognatha: *Aidanosagitta crassa*, *Eukrohnia hamata*, *Flaccisagitta lyra*, *F. scrippsae*, *F. enfrata*, *Parasagitta elegans*, *Spadella angulata*, and *Zono sagitta nagae*) have TTX stores or purported TTX-producing symbionts localized in the head near the raptorial apparatus [123,124] and, in the lab, immobilize copepods with their venom [125]. The ribbonworms (*Cephalothrix linearis* and *C.* sp.) also have high levels of TTX in the proboscis and may also use the toxin to subdue prey [105].

As in poison arrowworms and ribbonworms, the function of TTX in blue-ringed octopuses (*Hapalochlaena maculosa, H. fasciata, H. lunulata*, and *H.* sp. 1 [126]) was first inferred based on distribution. In these octopuses, TTX is prevalent in the posterior salivary glands [52,106,107, 127–129], the typical site of octopus venom [130]. Although the efficacy of the venom against the octopuses’ natural prey has not been quantified or compared to other octopus venoms directly, several human envenomations have been recorded (both lethal and sub-lethal) [131–136].

Finally, some carnivorous gastropods accumulate TTX as well as several paralytic shellfish poisons (PSPs), and Hwang *et al.* [137] suggest the snails might use these toxin stores offensively against prey. However, this tenet is untested. Dogwhelks (*Nucella lapillus*) and the Gulf Oyster Borer (*Thais haemastoma*) appear to inject paralytic muscle poisons into the gape between mussel and oyster valves, but the identity of the toxic agent was not determined [138,139]. Although serotonin and several small peptides have been indicated as major components of the Dogwhelk’s venom, they may not account for all symptoms [140–142]. Rovero *et al.* [139] observed similar symptoms to TTX or STX poisoning in the mussel, but further investigations have not yet been forthcoming.

## 4. Communicative Function of TTX

Several species have the ability to exploit TTX as a source of information. At least one species of puffer fish recognizes TTX as a pheromone. Sexually receptive male puffer fish (*Takifugu niphobles*) were attracted to less than 15 pM of TTX in a Y maze [112]. Sexually active females did not appear to recognize TTX or were not attracted to it at the concentrations tested. In the wild, ovulated oocytes have TTX stores in the vitelline envelope, which is the hypothesized site of release [112]. In *Utetheisa* moths, females detect and prefer males with higher quantities of pyrrolizidine alkaloids in their spermatophores; the alkaloids are subsequently invested into their clutch [143]. Whether male puffer fish preferentially fertilize better-defended (higher TTX content) eggs or are even induced to fertilize eggs by TTX as a cue remains untested. Additionally, insight on functionality may be gleaned by testing both males and females at additional reproductive stages. Further investigation of this nature is required to definitively assign the role of TTX as a sex pheromone in this species and to exclude alternative functional hypotheses about why TTX attracts these puffer fish. However, the broader implications of this hypothesis of TTX as a sex pheromone highlights the possibility that TTX may serve as an attractant in other species that endow their eggs with TTX (see above). No investigations of this nature, exclusive of this one species of puffer fish, have been conducted. Interestingly, surveys of TTX levels in puffer fish [10], blue-ring octopus (*Hapalochlaena fasciata*) [105], and a goby (*Yongeichthys criniger*) [144] revealed the toxin in the testes of mature males, though the ecological implications of this have not been explored.

Many TTX-accumulating snails (*Polinices didyma, Natica lineata, Natica vitellus, Zeuxis sufflatus, Niotha clathrata, Oliva miniacea, Oliva mustelina,* and *Oliva hirasei*) are attracted to the toxin, while non-toxic species are not attracted to TTX [30]. These snails appear to prefer foraging on TTX- or STX-laden food items [30]. The same species, except those of the genus *Oliva*, are also attracted to STX [145]. Snails that were most resistant to STX or TTX had stronger preference for the toxins and consequently exhibited higher toxicity [30,145]. Similarly, the puffer fish *T. rubripes* was attracted to TTX in spiked food items and thus appears to seek out TTX in its diet [31]. This behavior may have evolved if accumulating TTX or STX stores confer a selective advantage to the snails or puffer fish, such as defense from their own predators.

A parasitic copepod (*Pseudocaligus fugu*) also may be attracted to TTX, based on its specificity for toxic puffer fish hosts [146]. A higher number of parasites were found on fish with higher TTX quantities [146]. Another copepod (*Taeniacanthus* sp.) was discovered on *T. niphobles* but there was no difference in infection rates between fish with high and low levels of TTX [146]. These copepods accumulate TTX in the gut but not other tissues; the transitory nature of TTX in the gut suggests that TTX is not then sequestered for another purpose in the parasite [38]. However, the possibility of TTX-producing bacteria adhering to the carapace of the parasitic copepod suggests there may be more to this relationship [147]. Finally, although ecology of TTX in terrestrial taxa is not the focus of this review, it is worth noting that TTX serves as an alarm pheromone for larval newts (*Taricha torosa*) to avoid cannibalistic conspecifics [148].

## 5. Conclusions

The behavioral and chemical ecology mediated by TTX varies among organisms; TTX may serve as an antipredator defense, offensive weapon (venom), or for within- and between-species communication about the location of mates/eggs, potential food sources, or danger. Undoubtedly, a more complex picture will continue to emerge as further research is undertaken. Interestingly, one study revealed a boost in immune function via antibody and splenocyte proliferation in puffer fish administered a diet with TTX [149]. The mechanisms by which this was effected and the evolutionary significance of this pattern are unknown.

Tetrodotoxin is not only a major determinant in interactions limited to a few species, but certainly affects the ecology of entire communities, as do other marine natural products [150]. Zimmer and Ferrer [13] consider neurotoxins, such as TTX and STX, as keystone molecules. Although they may be produced by only a few members of an ecosystem, the bioaccumulation, potential symbiotic relationships, and co-opting of the toxin for other ecological functions in accumulating organisms reverberates far beyond the original producers. For example, the presence of STX in butter clams alters predatory behavior of well-known keystone species—otters—consequently altering benthic community assemblages [122]. As TTX spreads through trophic levels via bioaccumulation [151,152], ecosystem-altering events may occur. For example, populations of toxin-accumulating species may expand over undefended counterparts, or non-resistant apex predators may be poisoned through bioaccumulation––consequently triggering trophic cascades [13,92,122].

Given the multitude of organisms in which TTX has been identified, this brief synopsis highlights the shortage of matching ecological data. Although hypotheses can be proposed about functions in some species, the critical natural history data and empirical observations are completely bereft for other TTX-accumulating taxa. Studies on ecosystem level effects of TTX are also depauperate. Although an antipredator function of TTX is strongly suggested for many taxa, not one study demonstrates unequivocally that survival probability varies according to TTX content. The function of TTX in any microbial producers has not been tested. Secretion of TTX upon stimulation in puffer fish, snails, ribbonworms, and amphibians [54,64,95,96] may deter predators, but experiments with live predators for these species have not been completed. One species of puffer fish [99,100] supports a Batesian mimic and blue-ringed octopuses appear to be aposematic, but the protective moieties have not been definitively assigned to TTX. Eggs of another puffer fish were shown to be repellent to predators [116], but the TTX content of the eggs was not tested. Hatchery reared juvenile puffer fish were TTX-free and suffered higher predation than wild, TTX-laden, conspecifics [101]; however, maladaptive swimming behavior may confound interpretation of this data. Given that organisms (especially those that sequester) often rely on multiple defenses [27], the assumption that TTX confers any observed protection must be explicitly examined. Some fish appear to detect TTX [119–121], but whether this results in avoidance of TTX-laden food items needs confirmation. Thus, much work is needed to demonstrate an antipredator function of TTX. Similarly, the efficacy of TTX as a venom has not been sufficiently tested. While Ritson-Williams *et al.* [87] discovered that a Planocerid flatworm injects TTX into prey, there have been no demonstrations that injection of higher TTX quantities by predators translates to quicker immobilization of prey. Finally, TTX appears to attract [30,31,112,146] or repel [148] a variety of organisms and this invites a deeper examination of ecological functions. For example, further work examining the use of TTX as a sex pheromone is warranted and should be extended to other taxa. Even clear assumptions can be misleading [87] and experiments designed to test these hypotheses, whether such hypotheses are supported or disproved, will surely generate new insight and potential for new investigations into the ecology of TTX producers and accumulators.
